# Clonorchiasis in Patients with Biliary and Pancreatic Diseases: Diagnosis and Risk Factors

**DOI:** 10.1155/2020/2946541

**Published:** 2020-02-21

**Authors:** Guolin Liao, Huaqiang Ruan, Peng Peng, Shiquan Liu, Jianfu Qin, Zhihai Liang, Guodu Tang, Mengbin Qin, Jie'an Huang

**Affiliations:** ^1^Department of Gastroenterology, The Second Affiliated Hospital of Guangxi Medical University, Nanning 530007, China; ^2^Department of Gastroenterology, The First Affiliated Hospital of Guangxi Medical University, Nanning 530021, China

## Abstract

**Background:**

Many epidemiological studies have investigated the risk factors for clonorchiasis, but endoscopic findings of this disease in endoscopic retrograde cholangiopancreatography (ERCP) have not been well characterized. In this study, we evaluated clonorchiasis in ERCP in patients with biliary and pancreatic diseases.

**Methods:**

This was a retrospective two-center study in hospitalized patients who received ERCP between January 2012 and October 2018. All patients were divided into clonorchiasis and nonclonorchiasis groups. Data were analyzed using univariate analysis and multivariate analyses.

**Results:**

A total of 1119 patients were included, and clonorchiasis was diagnosed in 19.2% patients. Detection of *Clonorchis sinensis* eggs in bile samples was higher than that in fecal samples (85.9% vs. 58.7%; *P* = 0.001). In multivariate analysis, male patients (95% confidence interval (CI): 1.945–4.249, *P* = 0.001). In multivariate analysis, male patients (95% confidence interval (CI): 1.945–4.249, *P* = 0.001). In multivariate analysis, male patients (95% confidence interval (CI): 1.945–4.249, *P* = 0.001). In multivariate analysis, male patients (95% confidence interval (CI): 1.945–4.249, *P* = 0.001). In multivariate analysis, male patients (95% confidence interval (CI): 1.945–4.249, *P* = 0.001). In multivariate analysis, male patients (95% confidence interval (CI): 1.945–4.249,

**Conclusions:**

The detection of *C. sinensis* eggs was significantly higher in bile than in fecal samples; thus, bile samples represent a preferable sample for the diagnosis of clonorchiasis in patients with biliary obstruction. We found that male, age ≤ 60 years old, and CBD diameter < 12 mm were independent risk factors for clonorchiasis, while papilla fistula was a protective factor.*C. sinensis* eggs was significantly higher in bile than in fecal samples; thus, bile samples represent a preferable sample for the diagnosis of clonorchiasis in patients with biliary obstruction. We found that male, age ≤ 60 years old, and CBD diameter < 12 mm were independent risk factors for clonorchiasis, while papilla fistula was a protective factor.

## 1. Introduction

Clonorchiasis, which is caused by *Clonorchis sinensis*, globally affects more than 15 million people, 13 million of whom live in China and other parts of East Asia [1–3]. An investigation conducted from 2005 to 2014 in Guangxi Province, southern China, showed that the prevalence of clonorchiasis in the general population reached 9.9% [4]. Clonorchiasis causes mechanical and chemical injury, resulting in inflammation [5–7], obstruction [5–8], and cancerogenesis [1, 2, 9] in the intrahepatic and extrahepatic biliary tracts [8, 10]. Common assays for diagnosing clonorchiasis include serologic detection of parasite-specific antibody and DNA [11–15], egg detection in bile and fecal samples [16, 17], and imaging [18]. The detection of *C. sinensis* eggs in fecal samples is specific, but with low sensitivity [19, 20], and requires skillful technique [21], especially when the infection is mild or there is biliary obstruction. Endoscopic retrograde cholangiopancreatography (ERCP) is not only a method for angiography but also a safe and effective tool for the treatment of biliary and pancreatic disorders. *C. sinensis* eggs are detectable in bile [6, 7, 19], but few studies have compared the detection of *C. sinensis* between bile and fecal samples.

It is important to evaluate independent risk factors for clonorchiasis in a large cohort of patients. Although several general risk factors for clonorchiasis have been reported [22, 23], no study to date has examined the risk factors associated with endoscopic findings. Various biliary or pancreatic disorders require different endoscopic interventions. ERCP procedures are generally safe and effective, but endoscopists must have a thorough understanding of indications for the selected procedures. However, there are no published data that compare endoscopic manipulation with outcomes between patients with and without clonorchiasis.

This retrospective two-center study analyzed the diagnostic sensitivity by detecting *C. sinensis* eggs in bile and fecal samples and the risk factors associated with endoscopic procedures. Furthermore, endoscopic manipulation and outcomes were compared between patients with and without clonorchiasis.

## 2. Patients and Methods

### 2.1. Patients

Patients who had biliary or pancreatic disorders (including jaundice caused by biliary obstruction; clinical and biochemical or imaging data suggestive of biliary stones, tumors, and sclerosing cholangitis; pancreatic diseases including tumors, chronic pancreatitis, and pancreatic abscess; and pancreatitis of unknown etiology and sphincter of Oddi manometry) and received ERCP procedures between January 2012 and October 2018 at both the First and Second Affiliated Hospitals, Guangxi Medical University (Nanning, China), were included. Indications for ERCP followed the guidelines of the American Society of Gastrointestinal Endoscopy [24]. Patients were excluded if age < 18 years old, no detection of *C. sinensis* in both fecal and bile samples, prior ERCP, or loss of clinical data. This study protocol was approved by the Institutional Review Boards of both hospitals. Written informed consent was obtained from all participants.

### 2.2. Procedures

All ERCP procedures were conducted by well-trained and experienced endoscopists, who are certified to perform procedures of ERCP difficulty Grade 3 per the ERCP core curriculum [25]. The ERCP equipment involved a therapeutic duodenoscope (TJF-260V; Olympus Optical, Tokyo, Japan). Selective cannulation of the common bile duct (CBD) was performed by using a guidewire or standard catheter if patients had a preexisting sphincterotomy. All duodenoscopes were disinfected and decontaminated per the guidelines and confirmed by regular smear tests. Once guidewire cannulation was successfully established after duodenoscope entry, bile was aspirated by inserting a disposable 5 F standard sphincterotome catheter into the bile duct before injection of a contrast agent for the ERCP procedure. Approximately 2–8 mL of bile (average 4 mL) was collected from patients with a clinical diagnosis of cholangitis, as suggested by clinical manifestations (jaundice, fever, and right upper quadrant pain) or radiological manifestations of biliary obstruction. The aspirated bile was immediately transferred into a sterile tube. After the injection of contrast agent, the length of the widest part of the CBD was documented and the diameter, number, and position of any CBD stone were recorded. The endoscopist on site selected endoscopic procedures including cannulation, endoscopic sphincterotomy (EST), bile culture, endoscopic papillary balloon dilation (EPBD), bougie dilatation, basket, lithotripsy basket, balloon, brush, biopsy, stent implantation, and endoscopic nasobiliary drainage (ENBD) based on the patient's conditions and the Chinese guidelines for ERCP (2010). In this study, we used the formalin-ether concentration technique (FEC) to detect *C. sinensis* eggs in bile and/or feces for the pathogen diagnosis of clonorchiasis. The first detection of eggs in fecal samples was made before ERCP, and two more tests were repeated during hospitalization if the first detection was negative.

### 2.3. Observational Index

Biochemical and hematological markers were examined within 72 hours of admission before ERCP including leukocyte (white blood cell (WBC))/amylase (AMS)/total bilirubin (TBil)/direct bilirubin (DBil)/alanine aminotransferase (ALT)/aspartate transaminase (AST)/gamma glutamyl transpeptidase (GGT)/alkaline phosphatase (ALP)/carcinoembryonic antigen (CEA)/carbohydrate antigen 199 (CA-199). Demographics and clinical findings during hospitalization were collected including gender, age, endoscopic diagnosis, papilla types, CBD diameter, and CBD stone characteristics (shape, size, position, number, and color). The collection procedure and outcome data included the cannulation method, EST/EPBD/bougie dilatation/ENBD/stent implantation/brush/biopsy/bile culture, the cut size of the EST, basket/balloon/lithotripsy basket, bile culture results, brush results, immediate complications, post-ERCP pancreatitis (PEP), post-ERCP cholangitis (PEC), and serological baseline data. PEP was defined as having new or worsened abdominal pain for more than 24 h after persistent ERCP, accompanied by elevated serum amylase level more than three times the upper limit of normal. PEC was defined as having a fever > 38°C and lasting >24 h due to biliary causes after ERCP.

### 2.4. Statistical Analysis

Continuous variables were expressed as the mean and standard deviation or median and interquartile range, and differences were computed using the Student's *t*-test or nonparametric test. Categorical variables were analyzed by the Pearson's chi-square test or Fisher's exact test. The test level in univariate was unrestricted to 0.10 if the factors underscored the clinical importance. Multivariate regression analyses were used to identify independent risk factors. Logistic regression models were employed to calculate odds ratios with 95% confidence intervals (CIs). A two-tailed *P* value < 0.05 was considered statistically significant (SPSS 22.0 for Windows, SPSS, Chicago, IL, USA).

## 3. Results

### 3.1. Baseline Characteristics

**Figure 1:**
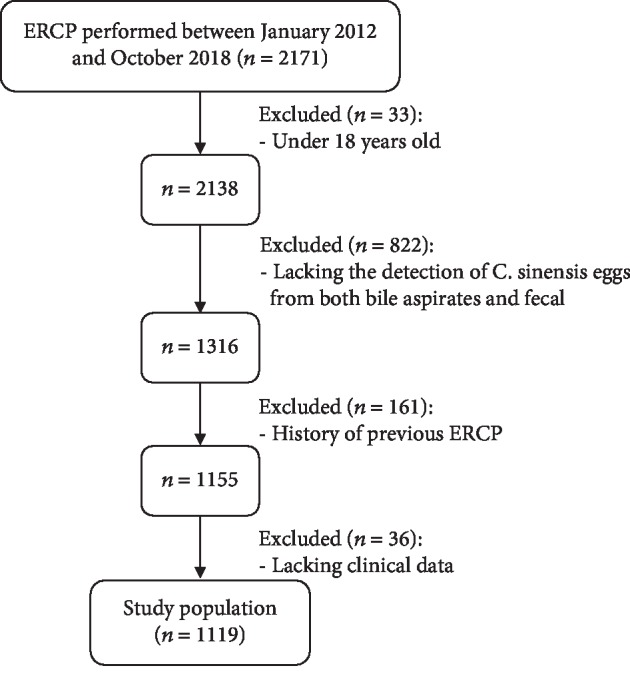
Flowchart of patients included in the study.

A total of 2171 consecutive patients who underwent ERCP in two hospitals between January 2012 and October 2018 were initially screened. Patients were excluded if age < 18 years old (*n* = 33), no detection of *C. sinensis* eggs in both bile and fecal samples (*n* = 822), prior ERCP (*n* = 161), or no clinical data (*n* = 36). Finally, 1119 patients were included and analyzed (Figure 1); 36.6% were female with a mean age of 57.2 ± 14.2 years (range: 20–92 years). Clonorchiasis was diagnosed in 19.2% of the 1119 patients as a result of detected *C. sinensis* eggs in the fecal and/or bile samples (Table 1).

### 3.2. Comparison of *C. sinensis* Egg Positivity in Bile and Feces

Among the 215 patients diagnosed with clonorchiasis, both bile and feces were collected from 92 patients for the detection of *C. sinensis* eggs. The eggs were detected in 85/92 (85.9%) bile samples, which was significantly higher than the 58.7% (54/92) detected in feces samples (*P* = 0.001), suggesting that the sensitivity of detecting eggs in bile was significantly higher than that in feces (Tables 1 and 2).

## 4. Risk Factors for Clonorchiasis

### 4.1. Univariate Analysis

Univariate analysis showed that gender, age (≤60 and >60), endoscopic diagnosis, papilla types, CBD diameter (<12 mm and ≥12 mm), and CBD stone shape were associated with clonorchiasis (*P* < 0.05, Table 3).

### 4.2. Multivariate Analysis

Multivariate logistic regression analysis showed that gender, age, endoscopic diagnosis, papilla type, and CBD diameter were independent risk factors for clonorchiasis. The clonorchiasis prevalence in male was 2.875 times higher than that in females (95% confidence interval (CI): 1.945–4.249, *P* = 0.0001). The clonorchiasis incidence in patients ≤ 60 years old was 1.732 times higher than that in patients > 60 years old (95% CI: 1.212–2.474, *P* = 0.003). Patients with papillary fistula were less susceptible to clonorchiasis compared to those with normal papilla (95% CI: 0.081–0.900, *P* = 0.033). However, minor papilla, papillary diverticulum, and papillary carcinoma did not correlate with clonorchiasis. Patients with CBD diameter < 12 mm had a 1.526-fold higher incidence compared to those with CBD ≥ 12 mm (95% CI: 1.093–2.130, *P* = 0.013). Although endoscopic diagnosis of clonorchiasis was significantly different between the two groups (95% CI: 3.774–84.822, *P* = 0.0001), other endoscopically diagnosed diseases were not (*P* > 0.05; Table 4).

### 4.3. Comparison of Endoscopic Procedures with Clonorchiasis Incidence

Univariate analysis showed that endoscopic procedures including the cannulation method, EST, ENBD, stent implantation, and balloon and bile culture were significantly associated with clonorchiasis (*P* < 0.05). Among the 215 patients with clonorchiasis, 202 received guidewire cannulation, 11 received dual guidewire, 1 received precut papillotomy, and 1 had failed cannulation. EST and ENBD were performed for removing stones, *C. sinensis* detection, or keeping bile drainage in 178 cases. Stent was implanted in 29 cases for biliary stenosis or for drainage (28 with single plastic stent and 1 with metal stent). Ballooning was used in 164 cases for dilating the bile duct. Bile was obtained in 150 cases for culture. Other procedures showed no correlation with clonorchiasis including bougie dilatation, brush, biopsy, the cut size of EST, basket, lithotripsy basket, bile culture results, brush results, immediate complications, PEP, and PEC (*P* > 0.05; Tables 5 and 6).

### 4.4. Analysis Characteristics of the CBD Stone

CBD stones in 562 cholelithiasis patients were nonsludge. Univariate analysis showed that size, location, number, and color of these stones were not significantly associated with clonorchiasis incidence (*P* > 0.05; Table 7).

## 5. Comparison of Biochemical and Hematological Findings between Patients with and without Clonorchiasis

The analyses showed that patients in both groups had elevated liver enzymes and jaundice. WBC, DBil, and ALT levels before ERCP in patients diagnosed with clonorchiasis were significantly higher than those in nonclonorchiasis patients (*P* = 0.001, 0.022, and 0.032, respectively). AMS, TBil, AST, ALP, GGT, CEA, and CA-199 levels showed no significant correlation with clonorchiasis (*P* > 0.05; Table 8).

## 6. Discussion

Clonorchiasis mainly occurs in East Asia and is associated with eating raw freshwater fish that carry the parasite [22]. Guangxi Province, where our patients resided, is part of southern China, and residents enjoy raw freshwater fish. In this study, clonorchiasis was diagnosed in 19.2% of the 1119 patients as a result of detected *C. sinensis* eggs in the fecal and/or bile samples. In addition, clonorchiasis mainly occurred in patients younger than 60 years old (155/215, 72.1%), with a 2.875-fold higher incidence in male than female patients. Clonorchiasis incidence in this cohort was significantly higher than that reported in the general Chinese population. Fang et al. [26] reported that clonorchiasis prevalence was 2.94% and 1.84% in males and females, respectively, with the highest prevalence found in the 50- to 59-year-old age group. Hoang et al. [27] reported that the prevalence in the male was 2.33 times higher than that in females in Vietnam, which supports our finding that more male clonorchiasis patients identified in southern China were susceptible to biliary or pancreatic disorders, including jaundice and elevated hepatic biochemical markers of TBil, DBil, ALT, AST, ALP, and GGT.

Detected *C. sinensis* eggs is direct evidence of clonorchiasis. The egg detection methods include Kato-Katz method (KK) and direct smear microscopy (DM) [18, 28]. The sensitivities of KK, FEC, and DM reported by Manuel et al. [29] were 71%, 50%, and 3%, respectively. The so-called “gold” standard combines the following four methods: KK, spontaneous sedimentation, FEC, and DM. However, Men et al. [19] referred to a combination of six KK plus two FECT methods as the “gold” standard. However, those standards involve multiple detection methods and a collection of consecutive fecal sample detection methods, which make them difficult to apply. Furthermore, when the infection activity is low or biliary obstruction is present, the probability of detecting eggs in fecal samples is extremely low. In our study, FEC was used for the detection of *C. sinensis* eggs in fecal and bile samples. We found that *C. sinensis* eggs were significantly more frequently detected in bile (79/92, 85.9%) than in fecal (54/92, 58.7%) samples, indicating that bile detection of *C. sinensis* eggs is preferable to fecal detection for the diagnosis of clonorchiasis in patients with biliary obstruction. Thus, bile detection should be incorporated into the established gold standards for diagnosing clonorchiasis.

Previous studies have identified male, eating raw fish, lower educational levels, and location of the villages as demographic or epidemiologic risk factors for clonorchiasis [22, 23, 30, 31]. Multivariate analysis showed that endoscopic diagnosis of clonorchiasis was significantly different between two groups, but it was not an independent risk factor for clonorchiasis. In addition, it could be influenced by subjective judgment (such as flocs and other parasites), resulting in a low sensitivity for direct diagnosis (15/41, 36.6%). Our research suggested both demographic factors of male and age ≤ 60 years old and CBD diameter < 12 mm as independent risk factors for clonorchiasis, while papilla fistula emerged as protective factor in the analysis. These findings are expected to identify a group of patients at high risk for clonorchiasis if they develop biliary or pancreatic disorders, especially in endemic regions.

When infected raw or undercooked fish is ingested by humans, the metacercariae excyst in the duodenum migrates into the intrahepatic bile ducts where eggs are laid. We wondered whether the excyst migration efficiency is related to papilla type. Our results showed that patients with normal papilla more frequently had clonorchiasis than patients with papillary fistula. As recently reported, positron emission tomography-computed tomography [32] can view the migration route within the host. This device may help investigate this issue among subjects with or without normal papilla.

We also compared the endoscopic procedures and outcomes between patients with and without clonorchiasis and found significant differences in conducting procedures including the cannulation method, EST, ENBD, stent implantation, balloon, and bile culture between the two groups. Patients with clonorchiasis were inclined to require guidewire cannulation, EST, ENBD, a single plastic stent, balloon, and bile culture. It is established that infection with *C. sinensis* is one of the most important factors for cholangiocarcinoma [33, 34], but our study found no significant differences in the brush results between clonorchiasis and nonclonorchiasis. The reason could be that patients with biliary or pancreatic disorders may not be representative; thus, future studies are needed to investigate the relationship between clonorchiasis and cholangiocarcinoma in a population without biliary or pancreatic disorders. There was no correlation between clonorchiasis and bile duct stones. As noted in this study, patients with clonorchiasis did not show special endoscopic features, so endoscopists must choose procedures after fully evaluating patients.

A few limitations of this study are worth mentioning. First, this was a retrospective study; thus, it may inevitably produce bias in data collection and selection. Prospective, large-cohort, multicenter studies are needed to confirm our findings. Second, only one assay (i.e., FEC) was used for the detection of *C. sinensis* in our study, and additional methods like KK were not used. Third, as bile samples were obtained during the ERCP procedure, the presence of *C. sinensis* eggs near the duodenal papilla may cause a positive bile finding, and ERCP procedures may have also increased the positive fecal finding. Fourth, the cohort we studied represented patients with biliary or pancreatic disorders, so these findings cannot be directly inferred to the general population.

## 7. Conclusions

In summary, our data showed that the detection of *C. sinensis* eggs was significantly higher in bile than in fecal samples; thus, the detection of *C. sinensis* in bile represents a preferable sample for the diagnosis of clonorchiasis in patients with biliary obstruction. We also found that male, age ≤ 60 years old, and CBD diameter < 12 mm were independent risk factors for clonorchiasis while papilla fistula as protective factor, indicating that patients with normal biliary or pancreatic tract are likely to be at high risk for clonorchiasis, in addition to age and gender, especially in endemic region.

## Figures and Tables

**Table 1:** Demographics and clonorchiasis in this cohort.

Study population	1119
Mean age (years, SD)	57.2 ± 14.2
Females	409 (36.6%)
Clonorchiasis	215 (19.2%)
*C. sinensis* eggs detected in feces	58.7%
*C. sinensis* eggs detected in bile	85.9%

**Table 2:** Comparison of egg detection between fecal and bile aspiration.

Eggs in bile aspiration	Eggs in feces		Total	*P*
	+	−		
+	41	38	92	0.001
−	13	0		

**Table 3:** Univariate analysis of risk factors for clonorchiasis.

Characteristic	*N* = 1119		*χ* ^2^	*P* value
	Clonorchiasis	Nonclonorchiasis		
Gender			42.93	0.0001
Male	178	532		
Female	37	372		
Age in years			23.58	0.0001
≤60	155	487		
>60	60	417		
Endoscopic diagnosis			56.26	0.0001
Cholelithiasis	152	624		
Malignant strictures	19	153		
Benign strictures	10	44		
Clonorchiasis	15	3		
Bile duct expansions for unknown reasons	7	35		
Pancreatic disorders	2	11		
Normal cholangiopancreatography	6	24		
Else	4	10		
Papilla types			21.10	0.0001
Normal	178	619		
Minor papilla	5	40		
Papillary carcinoma	2	43		
Papillary fistula	3	47		
Papillary diverticulum	27	155		
CBD diameter			24.13	0.0001
<12 mm	118	331		
≥12 mm	97	573		
CBD stone shape			10.60	0.001
Stone	94	468		
Sludge	58	156		

**Table 4:** Multivariate analysis of risk factors for clonorchiasis.

Factors	Wald	*P* value	OR	95% CI
Gender				
Male	28.080	0.0001	2.875	1.945-4.249
Female			1	
Age in years				
≤60	9.095	0.003	1.732	1.212-2.474
>60			1	
Endoscopic diagnosis				
Cholelithiasis	0.899	0.343	1.582	0.613-4.081
Malignant strictures	0.108	0.742	0.838	0.291-2.409
Benign strictures	0.007	0.932	1.051	0.329-3.358
Clonorchiasis	13.196	0.0001	17.892	3.774-84.822
Idiopathic bile duct expansions	0.191	0.662	1.321	0.379-4.606
Pancreatic disorders	0.088	0.767	0.763	0.127-4.588
Else	1.463	0.226	2.555	0.559-11.679
Normal cholangiopancreatography			1	
Papilla types				
Minor papilla	2.121	0.145	0.486	0.184-1.283
Papillary carcinoma	1.742	0.187	0.362	0.080-1.637
Papillary fistula	4.544	0.033	0.270	0.081-0.900
Papillary diverticulum	2.099	0.147	0.703	0.436-1.133
Normal papilla			1	
CBD diameter				
<12 mm	6.147	0.013	1.526	1.093-2.130
≥12 mm			1	

**Table 5:** Comparison of endoscopic procedures performed between clonorchiasis and nonclonorchiasis.

Characteristic	*N* = 1119		*χ* ^2^	*P* value
	Clonorchiasis	Nonclonorchiasis		
Cannulation method			10.47	0.011
Guidewire	202	850		
Dual guidewire	11	24		
Precut papillotomy	1	29		
Fail	1	1		
EST			13.55	0.0001
Yes	178	636		
No	37	268		
Cut size of EST			0.36	0.835
Big	4	11		
Medium	20	65		
Small	154	558		
EPBD			0.09	0.759
Yes	110	452		
No	105	452		
Bougie dilatation			1.93	0.165
Yes	10	66		
No	205	838		
ENBD			23.42	0.0001
Yes	178	595		
No	37	309		
Stent implantation			20.25	0.0001
Single plastic stent	28	189		
Metal stent	1	10		
Multiple stent	0	42		
No	186	663		
Basket			0.67	0.414
Yes	77	351		
No	138	553		
Balloon			17.45	0.0001
Yes	164	552		
No	51	352		
Lithotripsy basket			2.19	0.139
Yes	11	73		
No	204	831		
Bile culture			7.71	0.005
Yes	150	538		
No	65	366		
Brush			0.02	0.903
Yes	22	90		
No	193	814		
Biopsy			2.44	0.119
Yes	1	22		
No	214	882		

**Table 6:** Comparison of endoscopic procedure outcomes between clonorchiasis and nonclonorchiasis.

Characteristic	*N* = 1119		*χ* ^2^	*P* value
	Clonorchiasis	Nonclonorchiasis		
Bile culture results			2.94	0.086
Positive	39	181		
Negative	110	358		
Brush results			0.234	0.628
Malignant	3	16		
Benign	19	73		
Immediate complications			1.40	0.237
Yes	2	23		
No	213	881		
PEP			0.23	0.629
Yes	11	54		
No	204	850		
PEC			0.0001	1.000
Yes	1	4		
No	214	900		

**Table 7:** Analysis of characteristics of CBD stones between clonorchiasis and nonclonorchiasis.

Characteristic	*N* = 562		*χ* ^2^	*P* value
	Clonorchiasis	Nonclonorchiasis		
Stone size			3.26	0.196
≤5 mm	15	52		
≤15 mm	69	339		
>15 mm	10	77		
Stone position in CBD			9.76	0.135
Upper	17	80		
Intermediate	17	61		
Lower	35	159		
Upper & intermediate	13	44		
Lower & intermediate	7	75		
Upper & lower	1	19		
Dispersion	4	30		
Stone number			0.39	0.532
<3	65	308		
≥3	29	160		
Stone color			5.38	0.129
Yellow	16	57		
Black	5	24		
Brown	45	230		
White	2	1		

**Table 8:** Comparison of biochemical and hematologic findings between clonorchiasis and nonclonorchiasis.

Characteristics	Clonorchiasis	Nonclonorchiasis	*P* value
WBC (^∗^10^9^/L)	8.8 (6.6–11.2)	7.6 (5.6–9.9)	0.001
AMS	90.0 (52.0–193.3)	74.0 (51.0–123.0)	0.090
TBil (*μ*mol/L)	96.9 (26.6–171.9)	58.0 (20.1–160.2)	0.054
DBil (*μ*mol/L)	68.9 (16.4–126.9)	39.4 (10.1–117.3)	0.022
ALT (U/L)	87.0 (34.3–190.0)	67.0 (30.0–134.0)	0.032
AST (U/L)	65.0 (33.5–109.5)	53.0 (30.0–100.0)	0.086
ALP (U/L)	189.5 (120.3–284.5)	193.0 (118.0–334.0)	0.464
GGT (U/L)	310.5 (162.8–570.5)	297.0 (128.0–576.0)	0.890
CEA (ng/mL)	2.3 (1.4–3.0)	2.0 (1.2–3.6)	0.542
CA-199 (U/mL)	32.9 (10.3–139.1)	28.3 (9.0–133.4)	0.526

All data are represented by median and interquartile range (IQR).

## Data Availability

The data used to support the findings of this study are available from the corresponding author upon request.

## Abbreviations

ERCP: Endoscopic retrograde cholangiopancreatography
CBD: Common bile duct
EST: Endoscopic sphincterotomy
EPBD: Endoscopic papillary balloon dilation
ENBD: Endoscopic nasobiliary drainage
WBC: White blood cell
AMS: Amylase
TBil: Total bilirubin
DBil: Direct bilirubin
ALT: Alanine aminotransferase
AST: Aspartate transaminase
GGT: Gamma glutamyl transpeptidase
ALP: Alkaline phosphatase
CEA: Carcinoembryonic antigen
CA-199: Carbohydrate antigen 199
PEP: Post-ERCP pancreatitis
PEC: Post-ERCP cholangitis
FEC: Formalin-ether concentration technique
KK: Kato-Katz method
DM: Direct smear microscopy.
